# Evaluating the influence of financial investment in compulsory education on the health of Chinese adolescents: a novel approach

**DOI:** 10.1186/s12889-022-14125-5

**Published:** 2022-09-12

**Authors:** Kewen Yang, Shah Fahad, Feimin Yuan

**Affiliations:** 1 College of Economics and Management, Northwest A&F University, No.3 Taicheng Road, Yangling, 712100 Shaanxi China; 2 School of Management, Hainan University, Haikou, 570228 Hainan Province China; 3 College of Plant Protection, Northwest A&F University, No.3 Taicheng Road, Yangling, 712100 Shaanxi China

**Keywords:** Adolescent, Financial investment in compulsory education, Health, Heterogeneity, Influence mechanism

## Abstract

**Background:**

With China's aging and declining fertility rate, the importance of population quality is increasing. As the main force of the labor market in the future, the Chinese government tries to promote the development of adolescents by increasing the financial investment in compulsory education, so as to improve the future population quality of China and enhance the national competitiveness. Therefore, the aim of this study was to investigate the relationship between financial investment in compulsory education and the health of Chinese adolescents.

**Methods:**

This study specifically uses data obtained from China Education Panel Survey (CEPS). The data were nationally representative, which covered families, schools, and communities. For the CEPS data obtained, the 2013–2014 school year was the baseline, and two cohorts of 7th and 9th graders were the starting point of the survey. In the 2014–2015 school year, 8th-grade students who participated in the baseline survey (7th-grade students in the 2013–2014 school year) were followed up. Since the second period only began to investigate the data on financial investment in compulsory education, this article uses the data from the 2014–2015 academic year for research. OLS and Ordered Probit models were used to investigate the impact of financial investment in compulsory education on adolescent health.

**Results:**

With the doubling of financial investment in compulsory education, self-rated health increased by 0.021, frequency of illness decreased by 0.03, the number of sick leave days decreased by 0.207, and depression decreased by 0.191. The heterogeneity analysis shows that compared to only-child, high-income and nonagricultural groups, the financial investment in compulsory education has a greater impact on the health of adolescents with the characteristics of agriculture hukou, non-only-child and low-income families. Further analysis of the impact mechanism shows that financial investment in compulsory education exerts a significant influence on the health of adolescents by easing family budget constraints, improving school sports facilities, and increasing the expected return of health investment and social capital.

**Conclusions:**

Financial investment in compulsory education can enhance the health of adolescents, and vulnerable groups benefit more, which is conducive to promoting health equity.

**Supplementary Information:**

The online version contains supplementary material available at 10.1186/s12889-022-14125-5.

## Background

Since the reform and opening up, China's economy has developed rapidly, and in 2010, it became the second largest economy in the world. However, with the aging of the population and the continuous low fertility rate, the number of China's labor force declined for the first time in 2013, which means that the role played by the quantitative advantage of the population in China's future economic development is declining and the importance of population quality is increasing. As the main force of the future labor market, the investment effect of adolescents' human capital is related to the long-term development and destiny of the country [1]. Health is not only an important part of human capital, but also the basis for education, migration and other human capital to play its role. However, the current health status of adolescents is not optimistic. Research shows that the number of adolescents with depressive symptoms in China reaches 20.3% [2], which is much higher than 0.4–9.8% in other countries [3], and the detection rate is increasing year by year. Depression not only affects adolescents’ academic performance and peer relationships [3, 4], but also further affects future work performance and family life [5, 6] as depressive mood is prone to relapse [7]. In order to improve adolescent health, the Central Committee of the Communist Party of China and the State Council issued the Outline of Healthy China 2030 in 2016 and the Medium-and Long-Term Youth Development Plan (2016–2025) in 2017 to promote the healthy growth and development of adolescents from multiple dimensions such as health and education.

However, according to Grossman's theory of health capital demand, health is the result of many factors [8]. Existing research focuses on the influence of family socio-economic background [9], medical and health resources supply [10], public medical and health expenditure [11] and medical insurance [12] on adolescent health. However, as an important factor affecting adolescents' learning environment, few studies have investigated the impact of financial investment in compulsory education on adolescents' health [1].

Since 2000, especially after the implementation of the "new mechanism" in 2006, China's financial investment in basic education has continued to increase, which has promoted the rapid development of basic education. Before the early 1980s, the Chinese government was the sole supplier of basic education, with the central government responsible for providing financial expenditure and the local government responsible for the actual supply of basic education. However, with the continuous advancement of fiscal decentralization reform, the management system of "local government under the leadership of the State Council is responsible, graded management and county-oriented" has been implemented since 1985, which makes the main responsibility for the development of basic education fall to the county and township governments [13]. Because the funds mainly come from the tuition and miscellaneous fees paid by counties, townships, villages and families, the shortage of compulsory education funds and the unbalanced allocation of educational resources among regions are prominent [14]. In 2001, the weakening of rural and village financial resources caused by the reform of rural taxes and fees prompted the central government to carry out a major reform of the basic education management system, and the focus of financial investment in compulsory education was raised to the county-level government, forming a "county-based" system [13]. In December 2005, the State Council issued the Notice on Deepening the Reform of Rural Compulsory Education Funds Guarantee Mechanism (referred to as "New Mechanism"), which gradually incorporated rural compulsory education into public finance and greatly improved the public finance guarantee for compulsory education [15]. Calculated at comparable prices, in 2016, the average budgetary expenditure of primary school and junior high school students was 12.7 times and 11.3 times that of 2000, respectively. At the same time, the regional difference of average budgetary expenditure per student also showed a downward trend year by year. From 2006 to 2016, the coefficient of variation measuring the regional difference of average budgetary expenditure of primary school and junior high school students decreased from 0.76 and 0.44 to 0.42 and 0.31, respectively [16].

With the increase of financial investment in compulsory education, more and more scholars pay attention to its social and economic impact [14, 17–19]. However, the authors found that there are few studies on the influence of financial investment in compulsory education on adolescent health. In theory, the increase in financial investment in compulsory education is conducive to improving school sports facilities and promoting students to participate in physical exercise, thus directly improving their health. However, empirically, only Zhang et al. examined the health effects of financial investment in compulsory education [1]. Nevertheless, this paper only uses the provincial panel data to examine the correlation between them, it does not discuss the possible influence mechanism. This provides an opportunity for this paper to continue to study this problem. Therefore, this paper attempts to use the CEPS data of the 2014–2015 school year to deeply investigate the impact of financial investment in compulsory education on the health of Chinese adolescents. Specifically, this paper will examine the following three issues: First, does the financial investment in compulsory education affect the health of Chinese adolescents? Second, is there any difference in the impact among different groups? Third, what is the specific influence mechanism? The marginal contribution of this paper is mainly reflected in the following three aspects: First, because there are few literatures on the impact of financial investment in compulsory education on adolescent health from the perspective of public finance, the results of this study are conducive to a deeper understanding of the relationship between financial investment in compulsory education and adolescent development; Secondly, the analysis of the influence mechanism in this paper makes up for the deficiency of the existing research on the one hand, and provides a realistic basis for better guiding the financial investment in compulsory education to improve the health of adolescents on the other hand; Thirdly, under the background that human capital is increasingly important to the healthy development of China's future economy, the investigation of the health effect of financial investment in compulsory education is not only conducive to evaluating the value and significance of financial investment in compulsory education from a healthy perspective, but more importantly, it can improve adolescents' health from the perspective of financial investment in compulsory education and promote the healthy development of China's economy and society.

The rest is arranged as follows: the second part is theoretical hypothesis. The third part is research and design. The fourth part is the empirical results. The fifth part is further discussion. Finally, it is the conclusion and policy recommendations of this paper.

### Theoretical hypothesis

According to the theory of healthy capital demand, whether to invest in healthy capital depends on the cost and benefit of investment [8]. If the return is greater than the cost, it is beneficial to invest more healthy capital, so as to increase the healthy capital stock.

With the rapid improvement of public financial strength, the state's financial investment in compulsory education is increasing, and the impact of financial investment in compulsory education on adolescents' health is deepening. From the health point of view, the impact of financial investment in compulsory education on adolescent health is mainly manifested in the following four aspects.

First, the financial investment in compulsory education is conducive to alleviating the constraint of family budget and increasing parents' investment in adolescent health capital. Chinese families have a tradition of attaching importance to their children's education. In order to let their children receive a better education, they will spend a lot of money to let their children attend extra-curricular tutoring classes to improve their academic performance. With the increasing investment of parents in children's education, education expenditure has become the main item of household expenditure in China [14]. The increase of financial investment in compulsory education effectively reduces the burden of family education [15]. For example, Wu found that public education financial investment has a substitution effect on residents' education expenditure, and the lower the income, the stronger its substitution [17]. The above analysis shows that with the increase of financial investment in compulsory education, family education expenditure represented by tuition and fees will decrease, which is conducive to relaxing family budget constraints, increasing health capital investment for adolescents, and thus improving their health.

Second, the financial investment in compulsory education is conducive to improving school sports facilities, attracting young people to participate in physical exercise and directly improving their health. Sports facilities are an important material basis and guarantee for schools to carry out sports work, and play an important role in improving students' physical fitness. However, the completeness of school sports facilities is deeply influenced by the government's financial investment in education. With the increase of the Chinese government’s financial investment in compulsory education, the conditions for running schools in underdeveloped areas have been greatly improved [15]. From 2004 to 2018, the average sports field area of middle school students in the eastern, central and western regions has been increasing [20], which is conducive to the financial investment in compulsory education enhancing the health of adolescents by improving school sports facilities.

Third, the financial investment in compulsory education is conducive to improving the investment return of education and stimulating families to invest in the healthy capital of adolescents. Generally speaking, the inequality of education quality has the cumulative effect of advantages. Better basic education can often obtain longer years of education and have a greater probability of receiving higher education of higher quality [21]. With the increase of the Chinese government’s financial investment in compulsory education, on the one hand, education expenditure can improve the human capital investment of all families to their offspring; On the other hand, compared with the children of high-income families, the expenditure on education can promote the human capital investment of children from low-income families [18]. This means that the financial investment in compulsory education is beneficial to improve the educational yield. According to the theory of education investment, the longer the life, the longer the time limit of the income from education investment [22], the higher the expected income of health capital investment, which can promote health capital investment and improve adolescent health [8].

Fourth, the financial investment in compulsory education is conducive to improving the academic performance of adolescents, increasing their social capital and improving their health. The increasing in public education investment can significantly promote the gathering of high-quality teachers and outstanding students in schools [21]. Learning is the main task of students, and their grades affect their interpersonal relationships with teachers and classmates. Students with good grades can not only be loved by teachers by "benefiting" teachers, but also be loved by classmates by improving their academic performance through peer effect [23]. This helps to improve the quality of their social capital, make them feel comfortable at school, eat healthier, and report fewer cases of sleep difficulties, thus helping to improve their health [24].

On the basis of the above research, this paper holds that the financial investment in compulsory education can improve the health of adolescents, and the relaxation of family budget constraints, the improvement of school sports facilities, the enhancement of expected return of health investment and the increase of social capital play an important intermediary effect.

### Research design

#### Data and variables

China Education Panel Survey (CEPS) data are used in the present study. The data were designed and implemented by the China Survey and Data Center of Renmin University in China and are nationally representative. The survey covered families, schools, and communities. For the CEPS data obtained, the 2013–2014 school year was the baseline, and two cohorts of 7th and 9th graders were the starting point of the survey. A stratified sampling method was employed to randomly select 28 county-level units (counties, districts, and cities) across the country as survey sites, from which a total of 112 schools and 438 classes were randomly selected for the survey, and all students in the selected classes were enrolled. In the 2014–2015 school year, 8th-grade students who participated in the baseline survey (7th-grade students in the 2013–2014 school year) were followed up; 9449 people were successfully followed up, for a follow-up rate of 91.9%. Since the second period only began to investigate the data on financial investment in compulsory education, this article uses the data from the 2014–2015 academic year for research. In the data sorting process, sample points missing key variables, such as gender, were deleted. The final sample size of the present study is 6516.

The dependent variable studied in this paper is adolescent health. Health indicators can be roughly divided into objective and subjective indicators. Subjective indicators, though simple, have been shown to be predictive of mortality and disability [25]. In addition, subjective health indicators are more in line with the definition of health provided by the World Health Organization (WHO). This definition of health considers not only the absence of disease and good physical fitness, but also physical and psychological health and social welfare [26]. To more fully examine the impact of financial investment in compulsory education on the health of Chinese adolescents, this article uses both subjective and objective health indicators.

The indicators to measure subjective health include: (1) self-rated health based on the question "how is your overall health now?". The answers are "very bad, not very good, fair, relatively good and very good" represented by 1–5 respectively. (2) Depression, measured by asking about ten kinds of feelings in the past seven days, such as "unhappy, depressed, nervous, worried and sad". The answers are "never, rarely, sometimes, often and always" represented by 1–5 respectively. From the reliability test results, Cronbach's coefficient is 0.913, indicating that the scale has high reliability. From the validity test results, the average variance extraction (AVE) was used to measure the structural validity. The results showed that the AVE value was 0.574, which was greater than 0.5, indicating that the convergent validity passed the test. In summary, from the reliability and validity test results, this paper uses the above ten questions to measure "depression" is appropriate. This article adds the above ten questions to get the total value. Therefore, the greater the value, the more serious depression.

Indicators to measure objective health include: (1) the frequency of illness based on the question "have you often been ill in the past year?", with answers "no, rarely and often" represented by 1–3 respectively. (2) The number of sick leave days based on the question "How many days have you taken sick leave in the past year?" The larger the value, the more days off.

The main explanatory variable is the financial investment in compulsory education, which is expressed by public funds per student. Public investment in education mainly consists of educational expenses and investment in educational infrastructure. Among them, education expenditure is the main body of public education investment, which is used to develop various social education undertakings, especially compulsory education undertakings. Personnel funds and public funds are two major components of educational expenses. The former refers to teachers' wages, welfare and social security, etc. According to the policy of the Central Committee of the Communist Party of China and the State Council's Opinions on Comprehensively Deepening the Reform of Teachers' Team Construction in the New Era,^1^ the average wage level of compulsory education teachers is not lower than or higher than the average wage level of local civil servants, so there is little difference in personnel funds in the same region. The latter refers to the investment needed to ensure the normal operation of the school, which is an important basis for the local financial department to allocate funds to educational institutions according to the number of students in the plan. Since the CEPS database used in this paper only collects the indicators of public funds per student, referring to the methods of Jia and Liu [27], this paper uses public funds per student to measure the financial investment in compulsory education. In the questionnaire, according to the question "how much is the average financial allocation for junior middle school students this year?" to get. The data are provided by the school principal.

In addition, referring to the theory of health capital investment [8] and existing research literature [9], this paper also controls the demographic and sociological characteristics of adolescents, such as age, gender, hukou (i.e., residence registration), cognitive ability and whether they are in love or not; Family characteristics, such as whether the only-child, parents' marital status, whether parents quarrel,^2^ mother's education, family economic status; Community characteristics, such as health environment^3^; School characteristics, such as school level, which are provided by the school principal. Considering the differences in social and economic development level and medical resources in different regions, which may affect adolescent health, this paper further controls the fixed effect of districts and counties. Students in the same class often have many similarities, so clustering is carried out at the class level. With age, adolescents gradually develop a favorable impression of the opposite sex. However, because the romantic relationship between adolescents is relatively fragile, it is easy to affect the mood due to the breakdown of the relationship, resulting in health damage [28]. Therefore, this article controls for whether or not adolescents are in a love relationship. Compared with the harmonious relationship between parents, parents' frequent quarrel will lead to the adolescents' irritability and nervousness, which will damage their health [29]. Therefore, this paper controls whether parents quarrel or not. See Table 1 for the processing results, definitions of the variables and descriptive statistical results.

**Table 1:** Descriptive statistics of major variables

Variables	Meaning/value	Obs	Mean (%)	SD	Min	Max
**Dependent variables**						
Self-rated health	Five-category variable	6516	3.876	0.922	1	5
Illness frequency	Three-category variable	6516	1.969	0.448	1	3
Sick leave days	Continuous variable	6516	1.766	4.980	0	115
Depression	Same as above	6516	21.73	7.995	10	50
**Explanatory variables**						
Financial investment	Add 1 to take logarithm	6516	6.649	1.248	0	8.380
**Individual characteristics**						
Age	Continuous variable	6516	13.90	0.859	12	17
Gender	1, boy; 0, girl	6516	0.521	0.500	0	1
Hukou	1,agriculture; 0,nonagriculture	6516	0.528	0.499	0	1
Cognitive ability	Three-category variable	6516	2.046	0.810	1	3
Love	1, yes; 0, no	6516	0.114	0.318	0	1
**Family characteristics**						
Parental marital status	1, married; 0, not married	6516	0.925	0.263	0	1
Only child	1, yes; 0, no	6516	0.442	0.497	0	1
Whether parents quarrel	Same as above	6516	0.098	0.298	0	1
Mother's education	Years	6516	3.360	1.194	1	7
Family economic status	Five-category variable	6516	2.937	0.599	1	5
**Community characteristics**						
Health environment	Four-category variable	6516	2.786	0.624	1	4
**School characteristics**						
School level	Five-category variable	6516	4.033	0.799	2	5

Source: CEPS data for the 2014–2015 school year, the same hereinafter

Overall, Chinese adolescents have good health, with a mean self-rated health of 3.9, mean the frequency of illness of 2.0, mean the number of sick leave days of 1.8, and mean depression of 21.7. The results of the above health indicators are relatively consistent, indicating that the measurement of adolescents’ health status is relatively robust. The average logarithm of financial investment in compulsory education is 6.65, which is about 772 yuan per student.

#### Model specification

This paper focuses on examining the relationship between financial investment in compulsory education and adolescent health. Considering that the indicators to measure health include continuous variables, three-category variables and five-category variables, the basic econometric models of this paper are divided into the following two categories^4^:

First, for continuous variables, the ordinary least squares model is defined as follows:

1 $$Y_{i} = \alpha + \beta Finance_{i} + \gamma X_{i} + \mu_{{\text{c}}} { + }u_{i}$$

Second, for three-category and five-category variables, this article implements the following ordered probit model:

Assume that the range of the original value of a health variable is 1..., m; the ordered probit model can be expressed as

2 $$Y_{i} = j,if\;u_{j - 1} < Y_{i}^{*} < = u_{j} ,j = 1,\cdot\cdot\cdot,m$$

where $Y_{i}^{*}$ is a latent continuous variable behind the ordered categorical variable $Y_{i}$ and is affected by $Finance_{i}$, demographic and socio-economic variable $X_{i}$ and regional characteristics $\mu_{{\text{c}}}$:

3 $$Y_{i}^{*} = \beta Finance_{i} + \gamma X_{i} + \mu_{{\text{c}}} + u_{i} ,u_{i} \sim N(0,1)$$

In addition,$u_{0} = - \infty ,u_{j} < = u_{j + 1} ,u_{m} = \infty$.According to the assumpation of $u_{i}$ in Eq. (4), the probability that $Y_{i}$ takes a value of $j$ is:

4 $$P_{ij} = P(Y_{i} = j) = \Phi (u_{j} - \beta Finance_{i} - \gamma X_{i} - \mu_{{\text{c}}} ) - \Phi (u_{j - 1} - \beta IFinance_{i} - \gamma X_{i} - \mu_{{\text{c}}} )$$

where $\Phi (\cdot)$ is the cumulative density function of the standard normal distribution and $j = 1\sim 5$. If $\beta$ is positive, then as the explanatory variable increases, the probability of low-level values decreases and the probability of high-level values increases.

In these equations, *i* represents an adolescent and *Y* represents the health of adolescent *i*. *Finance* represents financial investment in compulsory education, *X* represents the control variable, *μ*_*c*_ represents the counties’ fixed effect, *u*_*i*_ is a random disturbance term, *β* and *γ* represent the coefficients of the corresponding variables, respectively.

### Empirical results and analysis

#### Benchmark model

According to the econometric model (1)-(4), this paper uses a variety of measurement models for estimation. The results are shown in Tables 2 and 3.

**Table 2:** Basic model results

Dep. variables	(1)	(2)	(3)	(4)
**Explanatory variables**	**Self-rated health**	**Illness frequency**	**Sick leave days**	**Depression**
**Main explanatory variable**				
Financial investment	0.021^**^	-0.030^***^	-0.207^***^	-0.191^**^
	(0.010)	(0.011)	(0.068)	(0.096)
**Individual characteristics**				
Age	-0.020	-0.017	0.195^*^	0.226
	(0.022)	(0.025)	(0.101)	(0.160)
Boy	0.125^***^	-0.277^***^	0.092	-0.386^**^
	(0.027)	(0.034)	(0.128)	(0.189)
Agriculture	-0.015	-0.069^*^	0.318^**^	-0.031
	(0.032)	(0.040)	(0.159)	(0.256)
Cognitive ability	-0.064^***^	0.067^***^	-0.220^***^	-0.253^*^
	(0.018)	(0.019)	(0.084)	(0.138)
Love	-0.067	0.025	0.908^***^	2.842^***^
	(0.042)	(0.057)	(0.255)	(0.357)
**Family characteristics**				
Parents married	0.114^**^	0.032	-0.709^**^	-0.762^**^
	(0.049)	(0.066)	(0.288)	(0.378)
Only child	0.015	0.022	0.109	-0.625^***^
	(0.033)	(0.037)	(0.153)	(0.235)
Parents quarrel	-0.323^***^	0.356^***^	0.104	4.187^***^
	(0.049)	(0.061)	(0.201)	(0.368)
Mother's education	0.007	-0.002	0.202^*^	-0.023
	(0.016)	(0.018)	(0.103)	(0.118)
Family economic status	0.206^***^	-0.089^***^	0.004	-1.020^***^
	(0.026)	(0.028)	(0.199)	(0.206)
**Community characteristics**				
Health environment	0.108^***^	-0.066^**^	-0.257^*^	-0.825^***^
	(0.023)	(0.030)	(0.133)	(0.165)
**School characteristics**				
School level	-0.027	0.032	-0.001	0.595^***^
	(0.025)	(0.024)	(0.093)	(0.210)
Constant	-	-	2.134	25.681^***^
	-	-	(1.806)	(3.126)
County (District) FE	Y	Y	Y	Y
Observations	6516	6516	6516	6516
Wald/F statistic	537.54	211.08	5.937	15.440
Pseudo-R^2^/Adj- R^2^	0.025	0.025	0.025	0.079

Note: Values in parentheses are the cluster robust standard error. *p < 0.1, **p < 0.05, ***p < 0.01. Due to space limitations, the cut point is omitted

**Table 3:** Marginal effects in the ordered probit model

Explanatory variables	Y = 1	Y = 2	Y = 3	Y = 4	Y = 5
Financial investment	**Self-rated health**				
	-0.0004^**^	-0.002^**^	-0.005^**^	0.001^**^	0.007^**^
	(0.0002)	(0.001)	(0.002)	(0.0003)	(0.003)
	**Illness frequency**				
	0.006^***^	-0.001^**^	-0.005^***^	-	-
	(0.002)	(0.001)	(0.002)	-	-

Note: Values in parentheses are the Delta-method standard errors

^*^p < 0.1, **p < 0.05, ***p < 0.01

Table 2 shows that financial investment in compulsory education exerts a significant influence on adolescents’ health. Specifically, with the doubling of financial investment in compulsory education, self-rated health increased by 0.021 units in the probit model, frequency of illness decreased by 0.03 units in the probit model, the number of sick leave days decreased by 0.207, and depression decreased by 0.191. These results suggest that financial investment in compulsory education has a significant positive impact on adolescents’ health, which is consistent with the existing research results [1].

Since self-rate of health and frequency of illness are categorical variable, the estimated coefficients in Table 2 only reflect the relative impact of financial investment in compulsory education, not the marginal effect. Therefore, the marginal effects of financial investment in compulsory education on self-rated health and frequency of illness are examined in combination with the estimates of each cut-off point. The results are shown in Table 3. It can be seen from the table that the probability of classifying self-rated health as very unhealthy, relatively unhealthy and fair decreases by 0.04%, 0.2% and 0.5% for every doubling of financial investment in compulsory education, and the probability of being classified as relatively healthy and very healthy increases by 0.1% and 0.7% respectively. The probability of classifying frequency of illness as no increases by 0.6%, and the probability of being classified as rare and frequent decreases by 0.1% and 0.5%.

According to the regression results, the control variables in Table 2 essentially match the theoretical expectations. In terms of individual characteristics, boy are healthier than girl. Compared with non-agricultural hukou, adolescents with agricultural hukou have a lower frequency of sickness and higher sick leave days. This may be because rural adolescents often do farm work, which leads to better physical fitness. However, due to nutritional and medical conditions, recovery from illness is relatively slow [30]. The higher the cognitive ability, the worse the self-rated health and the higher the frequency of illness, but the number of sick leave days will be reduced and the depression will be relieved. This may be due to the rough measurement of intelligence in this paper, which leads to the unstable relationship between the two. Adolescents fall in love too early, which will lead to distraction of energy, affect health investment, and is not conducive to improving health [28]. In terms of family characteristics, compared to divorced parents, married parents can provide more resources for adolescents and improve their health. Compared with non-only children, only children has lower depression, which may be due to the fact that only children receives more care from their parents and elders, which accords with the theory of resource dilution. Compared with parents' harmonious relationship, parents' frequent quarrels will lead to adolescents' irritability and nervousness, thus damaging their health [29]. Mothers with higher education know how to improve their children’s health on the one hand, on the other hand they have higher expectations of their children, which makes their health worse [9]. The better the family's economic conditions, the better the ability to invest in the health of adolescents, so as to improve their health. In terms of community environment, the better the community health environment is, the better it is for adolescents to get clean water and clean air, thus improving their health. In terms of the school characteristics, the better the school, the richer the sports facilities, and the better it is for improving the health of adolescents. However, this paper finds that the better the school, the more serious the depression of students, but it does not affect other health indicators, which may be due to the rough data to measure the school quality.

### Two-stage least squares (2SLS) method

The use of different health variables showed that financial investment in compulsory education has a significant positive impact on adolescents’ health, but we cannot fully exclude endogeneity problems caused by reverse causality and the omission of variables. In terms of reverse causal effect, if adolescents' health is poor, it will promote the government's financial investment in schools to improve adolescents' health by implementing nutrition plans [1], which leads to underestimation of the health effect of financial investment in compulsory education. In terms of omitted variables, schools and families are the main activity fields of adolescents, and both of them have the willingness to actively intervene in their health. If it is expected that schools will actively intervene in adolescents' health, under the constraint of limited family budget, this may prompt parents to allocate more scarce resources such as income to adolescents' study such as extracurricular training, which will lead to a decline in family investment in adolescents' health, thus reducing the health effect of financial investment in compulsory education [31]. Therefore, this article made additional effort to select control variables. To reduce the problem of omitted variables caused by unobservable factors, it is necessary to control for the relevant influencing factors, such as demographic and sociological characteristics, family background, school environment, and the fixed effect of districts and counties as much as possible. Furthermore, the article searches for instrumental variables of financial investment in compulsory education to reduce the bias and inconsistency caused by possible endogeneity.

An effective instrumental variable must satisfy two conditions: it is related to the endogenous variable and it is not related to the random disturbance term. In this article, whether students with hukou in other counties and districts also have public funds (referred to as instrumental variable, for short) is used as an instrumental variable of financial investment in compulsory education.

On the one hand, whether students with hukou in other counties and districts also have public funds reflects whether the school funds are sufficient, which is related to the financial investment in compulsory education obtained by the school [19]. The test results show that there is a significant correlation between them, and the results are shown in Table 4. As seen from the table, regardless of whether other variables and the fixed effect of districts and counties are controlled for, the instrumental variable is significantly positively correlated with financial investment in compulsory education, and the coefficient is relatively stable. That is, the instrumental variable is correlated with the endogenous variables, so the correlation hypothesis for the instrumental variable is satisfied. In addition, when other variables and the fixed effect of districts and counties are controlled for, the F-statistic value is greater than 10, indicating that there is no weak instrumental variable problem [32].

**Table 4:** Instrumental variable test (1): Correlation test

Dep. variables	Financial investment		
**Explanatory variables**	**(1)**	**(2)**	**(3)**
Instrumental variable	0.875^***^	0.921^***^	1.084^***^
	(0.314)	(0.340)	(0.396)
Control variables	N	Y	Y
County (District) FE	N	N	Y
Observations	6272	6272	6272
F statistic	7.779	3.942	175.953
Adj- R^2^	0.087	0.135	0.363

Note: (1) Values in parentheses are cluster-robust standard errors, *** *p* < 0.01, ** *p* < 0.05, * *p* < 0.1. (2) To save space, neither control variables nor constants are reported here. (3) The controlled variables are the same as those in Table 2

On the other hand, whether instrumental variables can directly affect adolescent health is largely affected by the type of school and the number of students from other counties. Generally speaking, the better the school is, the more likely it is for students from other counties to get financial investment, thus directly affecting the health of adolescents. Therefore, this paper controls the school level to reduce the direct impact of instrumental variables on adolescent health. In addition, the number of students from other counties and districts may also affect the amount of financial investment received by schools, and then affect the health of adolescents. If there are a large number of students with hukou in other counties and districts, there will be more investment in funds, which may attract more students from other counties and districts to study here, thus affecting the health of adolescents, otherwise it will be difficult to have a direct impact. According to the analysis of the data of students with different hukou types in CEPS database, it can be found that the proportion of schools with more than 70% of students with local hukou is 77.7%, which means that even if students from other counties also have financial investment, the financial investment that schools can get is relatively limited because there are fewer students from other counties. Therefore, this paper argues that the instrumental variable cannot directly affect adolescent health. According to the existing research, this instrumental variable is exogenous and has been used by scholars [19].

Considering that there is only one instrumental variable, which has the characteristics of just identification, in order to test the exogenous of the instrumental variable, this article uses the method of Conley et al. for reference to test the exogenous of the instrumental variable [33], and the results are shown in Table 5.

**Table 5:** Instrumental variable test (2): Exogeneity test

Dep. variables	(1)	(2)	(3)	(4)
**Explanatory variables**	**Self-rated health**	**Illness frequency**	**Sick leave days**	**Depression**
**Part A**				
Instrumental variable	0.071^*^	0.018	-0.589^**^	-0.148
	(0.039)	(0.015)	(0.241)	(0.323)
Control variables	Y	Y	Y	Y
County (District) FE	Y	Y	Y	Y
Observations	6412	6412	6412	6412
F statistic	12.376	5.687	6.139	13.832
Adj- R^2^	0.057	0.026	0.022	0.075
**Part B**				
Financial investment	0.009	-0.014^***^	-0.164^**^	-0.201^*^
	(0.010)	(0.004)	(0.075)	(0.104)
Instrumental variable	0.062	0.034^**^	-0.412	0.070
	(0.044)	(0.015)	(0.251)	(0.338)
Control variables	Y	Y	Y	Y
County (District) FE	Y	Y	Y	Y
Observations	6412	6412	6412	6412
F statistic	12.535	5.836	6.753	14.824
Adj- R^2^	0.057	0.026	0.023	0.075

Note: (1) Values in parentheses are cluster-robust standard errors, *** *p* < 0.01, ** *p* < 0.05, * *p* < 0.1. (2) To save space, neither control variables nor constants are reported here. (3) The controlled variables are the same as those in Table 2

As seen from part A of the Table, when the financial investment in compulsory education is not controlled for, the instrumental variable has a significant impact on self-rated health and the number of sick leave days indicators. After controlling for the financial investment in compulsory education, as shown in part B, the instrumental variable has a significant impact on only the frequency of illness indicator and does not affect other indicators measuring the health of adolescents. Therefore, in general, the instrumental variable is exogenous relative to the health of adolescents. Notably, the instrumental variable does not meet the exogeneity requirements for the frequency of illness indicator. Therefore, in the 2SLS regression analysis, the relationship between the financial investment in compulsory education and this indicator is temporarily not considered.

The rationality of the instrumental variable has been tested in detail and fully, which shows that the instrumental variable selected in this paper are suitable. In this section, the instrumental variable selected above will be used for model estimation. The results are shown in Table 6.

**Table 6:** 2SLS regression analysis

Dep. variables	(1)	(2)	(3)	(4)
**Explanatory variables**	**Self-rated health**	**Illness frequency**	**Sick leave days**	**Depression**
Financial investment	0.066	0.017	-0.544^**^	-0.137
	(0.041)	(0.017)	(0.248)	(0.289)
Control variables	Y	Y	Y	Y
County (District) FE	Y	Y	Y	Y
Observations	6272	6272	6272	6272
F statistic	14.414	4.933	5.212	14.526
Adj- R^2^	0.052	0.021	0.020	0.079
DWH test	1.97	5.06^*^	2.7	0.04

Note: (1) Values in parentheses are cluster-robust standard errors, *** *p* < 0.01, ** *p* < 0.05, * *p* < 0.1. (2) To save space, neither control variables nor constants are reported here. (3) The controlled variables are the same as those in Table 2

First, the endogeneity of financial investment in compulsory education was tested. The Durbin-Wu-Hausman (DWH) test indicated failure to reject the null hypothesis for all of the health indicators except the frequency of illness indicator. Therefore the endogeneity has been accounted for by the selection of control variables and the estimation results of the baseline regression are credible.

Although no endogeneity problem in financial investment in compulsory education is detected, the estimation results of the instrumental variable show that financial investment in compulsory education has a significant impact on the number of sick leave days, which indicates that the impact of financial investment in compulsory education on adolescents’ health is robust.

Due to the difficulty of directly using the instrumental variable method to estimate the ordered probit model, this article adopts linear 2SLS. Following the treatment specified above, the extent of the impact of financial investment in compulsory education on adolescents’ health in the basic model and the instrumental variable model are no longer comparable. However, the focus of this article is the nature of the impact, and in this regard the results are consistent.

In summary, the estimation results of the baseline regression and 2SLS regression show that financial investment in compulsory education has a significant positive impact on the health of adolescents in China.

### Further analysis

#### Heterogeneity analysis

The fourth part discusses the relationship between the financial investment in compulsory education and adolescent health in detail, but does not answer the heterogeneity of the impact of the financial investment in compulsory education on different groups. Both family and government investment are important ways to improve adolescent health. With the continuous increase of government financial investment in education, to a certain extent, it can make up for the shortage of family health investment, thus narrowing the health differences among adolescent groups and promoting health equity. Income is the key factor affecting family health investment [8]. The income difference is mainly reflected in the urban–rural difference at the macro level, and it is affected by the dilution of family resources by the number of children at the family level. Therefore, this part examines the heterogeneous effects of financial investment in compulsory education on adolescent health from different angles, such as hukou, number of children and income, in order to test whether the financial investment in compulsory education is beneficial to make up for the shortage of investment in adolescent health by disadvantaged families, enhance adolescent health of disadvantaged families, narrow the health differences among different adolescent groups, and finally promote health equity. Table 7 reports the estimated results.

**Table 7:** Heterogeneity analysis

Dep. variables	Hukou		Only child		Family economic status	
	**(1)**	**(2)**	**(3)**	**(4)**	**(5)**	**(6)**
	**Agriculture**	**Nonagriculture**	**Yes**	**No**	**Rich**	**Poor**
**Self-rated health**	0.021^**^	0.004	-0.013	0.037^***^	-0.005	0.018^**^
	(0.010)	(0.019)	(0.014)	(0.013)	(0.027)	(0.008)
**Illness frequency**	-0.011^**^	-0.013	-0.020	-0.039^**^	-0.030^***^	-0.010^***^
	(0.004)	(0.008)	(0.017)	(0.015)	(0.010)	(0.004)
**Sick leave days**	-0.252^***^	-0.101	0.078	-0.352^***^	-1.393	-0.150^**^
	(0.088)	(0.097)	(0.093)	(0.054)	(0.951)	(0.059)
**Depression**	-0.200^**^	-0.149	-0.235^*^	-0.170	-0.092	-0.190^**^
	(0.093)	(0.160)	(0.133)	(0.109)	(0.352)	(0.091)

Note: (1) Values in parentheses are cluster-robust standard errors, *** *p* < 0.01, ** *p* < 0.05, * *p* < 0.1. (2) To save space, neither control variables nor constants are reported here. (3) The controlled variables are the same as those in Table 2

For the heterogeneity at the hukou level, column (1) of Table 7 shows that the financial investment in compulsory education has a significant effect on the health indicators of adolescents with agricultural hukou; Column (2) of Table 7 shows that the financial investment in compulsory education has no significant impact on the health of adolescents with non-agricultural hukou.

As for the heterogeneity of the number of children, column (3) of Table 7 shows that the financial investment in compulsory education only has a significant impact on the depression of adolescents from only-child families, but does not affect other health indicators; Column (4) of Table 7 shows that except for the depression, the financial investment in compulsory education has a significant impact on other health indicators of adolescents from non-only-child families.

For the heterogeneity of income level, this paper classifies those whose family economic conditions are "very difficult, relatively difficult and medium" into one category, which is used to indicate that the family economic conditions are poor; "Relatively rich and very rich" are classified into one category, which is used to indicate that the family has better economic conditions. Column (5) of Table 7 shows that the financial investment in compulsory education only has a significant impact on the disease frequency of adolescents from families with better economic conditions, but does not affect other health indicators; Column (6) of Table 7 shows that financial investment in compulsory education has a significant impact on all health indicators of adolescents from families with poor economic conditions.

The above results show that there are significant differences in the impact of financial investment in compulsory education on adolescent health in different hukou, number of children and income. Compared with non-agricultural hukou, only-child and high-income groups, the financial investment in compulsory education has a greater impact on the health of adolescents with the characteristics of agriculture hukou, non-only-child and low-income families, which is conducive to improving the health of adolescents from disadvantaged families, narrowing the health differences among different adolescent groups and promoting health equity.

### Influential mechanism analysis

This part further discusses the possible mechanism that the financial investment in compulsory education affects adolescent health. According to the theoretical analysis, firstly, the financial investment in compulsory education can increase parents' health capital investment in adolescents by alleviating family budget constraints. This paper uses the indicator of "parents and adolescents watch sports events together" to measure. Secondly, the financial investment in compulsory education can directly impact their health by improving school sports facilities. This paper uses the indicator of "school playground allocation" to measure. Third, the financial investment in compulsory education can impact the expected return of education investment, so as to stimulate the family's health capital investment in adolescents. This paper uses the indicator of "parental education expectation" to measure, which is because parents are not only rational people, but also the main decision-makers of adolescent education investment. The parental educational expectation reflects their expectation of the return on future education investment. The higher return on future education investment makes them more likely to invest in adolescent health, so as to prolong the payback period of education investment. Finally, financial investment in compulsory education can impact adolescents' health by improving their social capital. This paper uses the indicator of "number of learning good friends" to measure. The above four indicators can be obtained directly from the CEPS data of 2014–2015 school year, and will not be described in detail.

The mediating effect model in this paper includes linear model and nonlinear model, and the Karlson-Holm-Breen (KHB) method proposed by Breen et al. can be used for mediating effect analysis of linear model and nonlinear model, and is also suitable for multidimensional mediating variables [34]. Therefore, this paper uses KHB method to test whether the above four intermediate variables are the channels through which the financial investment in compulsory education affects adolescents' health.

**Fig. 1:**
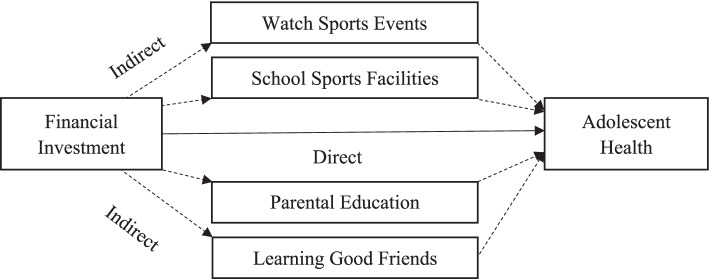
Mechanism of financial investment in compulsory education

Figure 1 shows the specific mechanism of financial investment in compulsory education affecting adolescent health. According to Fig. 1, the impact of financial investment in compulsory education on adolescent health through four different paths is called indirect effect, and the impact of financial investment in compulsory education on adolescent health without any path is called direct effect. The sum of indirect effect and direct effect is the total effect of financial investment in compulsory education on adolescent health.

The test results of KHB method are reported in Table 8. From the total effect, the financial investment in compulsory education can promote the enhance of adolescents' self-rated health, the decrease of illness frequency, the reduction of sick leave days and depression. In terms of direct effect, financial investment in compulsory education has a significant impact on indicators such as illness frequency and sick leave days, but does not affect indicators such as self-rated health and depression. In terms of indirect effect, the mediating effect is significant in the self-rated health and depression model, but not in the illness frequency and sick leave days model. The above results show that, on the one hand, in the self-rated health and depression model, the total effect and indirect effect are significant, but the direct effect is not significant, which means that "parents and adolescents watch sports events together, school sports facilities, parents' educational expectation and the number of learning good friends" play an important mediating effect in the process of financial investment in compulsory education influencing adolescents' self-rated health and depression. On the other hand, in the model of sick frequency and sick leave days, the total effect and direct effect are significant, but the indirect effect is not significant, which means that the mediation effect of the above four intermediary variables is limited in the process of reducing the sick frequency and sick leave days of adolescents by financial investment in compulsory education.

**Table 8:** Mediating effect test based on KHB method

Variables	(1)	(2)	(3)	(4)
	**Self-rated health**	**Illness frequency**	**Sick leave days**	**Depression**
Total effect	0.017^**^	-0.011^***^	-0.207^***^	-0.191^*^
	(0.008)	(0.004)	(0.065)	(0.098)
Direct effect	0.010	-0.009^**^	-0.214^***^	-0.091
	(0.009)	(0.004)	(0.072)	(0.099)
Indirect effect	0.007^**^	-0.001	0.007	-0.101^***^
	(0.003)	(0.001)	(0.018)	(0.032)
Proportion of indirect effects (%)	39.81	11.87	-3.13	52.63
Watch sports events	13.44	-15.00	22.66	10.70
School sports facilities	36.32	113.28	328.19	55.52
Parental education expectation	26.46	-18.17	-200.91	17.05
Learning good friends	23.78	19.90	-49.94	16.72

Note: (1) Values in parentheses are cluster-robust standard errors, *** *p* < 0.01, ** *p* < 0.05, * *p* < 0.1. (2) To save space, neither control variables nor constants are reported here. (3) The controlled variables are the same as those in Table 2

In addition, the above results are robust in terms of the proportion of indirect effects. In the model of self-rated health and depression, the indirect effects accounted for 39.18% and 52.63%, respectively, which indicated that the above four intermediary variables played an important intermediary effect in the process of impacting adolescents' self-rated health and depression by financial investment in compulsory education [35]. In the model of sick frequency and sick leave days, the indirect effect accounts for only 11.87% and -3.13%, respectively, which indicates that the above four intermediary variables have limited mediating effects in the process of reducing the sick frequency and sick leave days of adolescents by financial input in compulsory education [35].

Judging from the proportion of mediation effect of each intermediary variable, different indicators of intermediary variables play different roles. In the self-rated health model, "school sports facilities" has the greatest mediating effect, reaching 36.32%; The mediating role of "parents' educational expectation" ranks second, accounting for 26.46%; The intermediary role of "the number of learning good friends" ranks third, accounting for 23.78%; The mediating effect of "parents and adolescents watch sports events together" is the smallest, accounting for 13.44%. In the depression model, "school sports facilities" has the greatest mediating effect, reaching 55.52%; The mediating role of "parents' educational expectation" ranks second, accounting for 17.05%; The intermediary role of "the number of learning good friends" ranks third, accounting for 16.72%; The mediating effect of "parents and adolescents watch sports events together" is the smallest, accounting for 10.70%.

On the whole, the above results show that "parents and adolescents watch sports events together, school sports facilities, parents' educational expectation and the number of learning good friends" play an important intermediary role in the process of impacting adolescents' health by financial investment in compulsory education. Among them, the mediating effect of "school sports facilities" is the biggest, the mediating effect of "parents' educational expectation" is the second, the mediating effect of "the number of learning good friends" is the third, and the mediating effect of "parents and adolescents watch sports events together" is the smallest.

## Discussion

With China's aging and declining fertility rate, the importance of population quality is increasing. Adolescents as the main force of the future labor market, China's financial investment in compulsory education is increasing, trying to improve the population quality and enhance the national competitiveness by promoting the development of adolescents. However, through the review of the existing literature, there are few studies to investigate the impact of financial investment in compulsory education on adolescent health. Therefore, this paper uses the CEPS data of 2014–2015 school year to investigate the relationship between financial investment in compulsory education and adolescent health. The results show that financial investment in compulsory education can significantly impact the health of adolescents. Considering the possible endogeneity problem, the results are still robust after selecting appropriate instrumental variables to deal with endogeneity. The heterogeneity analysis shows that there are significant differences in the impact of financial investment in compulsory education on adolescent health in terms of hukou, number of children and income. Compared with non-agricultural, only-child and high-income groups, the financial investment in compulsory education has a greater impact on the health of agriculture hukou, non-one-child and low-income groups, which helps to narrow the health differences among different adolescent groups and promote health equity. Further analysis of the impact mechanism shows that the financial investment in compulsory education can impact adolescent health through the following four different channels. Firstly, the financial investment in compulsory education can increase parents' health capital investment in adolescents by alleviating family budget constraints. Secondly, the financial investment in compulsory education can directly impact their health by improving school sports facilities. Third, the financial investment in compulsory education can impact the expected return of education investment, so as to stimulate the family's health capital investment in adolescents. Finally, financial investment in compulsory education can impact adolescents' health by improving their social capital.

Dunn et al. argues that the local government's fiscal expenditure can provide a more favorable environment for residents and enable them to obtain more available resources, which is equivalent to increasing their real income, thus alleviating the negative impact of income inequality [36]. Their subsequent empirical research on the United States did find that income inequality would lead to an increase in mortality in the United States. When the local government's financial expenditure on education increases, the impact of income inequality on mortality will decrease. The study of Dunn et al. is different from this article [36]. On the one hand, they examine the overall educational financial investment including basic education and higher education; on the other hand, they examine adults. However, the research conclusions of Dunn et al. is consistent with the research conclusion of this paper on Chinese adolescents, and both found that educational financial investment can improve health [36]. To a certain extent, this shows that the research results of this paper are robust.

### Limitations

Although this paper discusses the relationship between financial investment in compulsory education and adolescent health, and obtains new findings, there are still some shortcomings in this paper that need to be improved due to the availability of data. On the one hand, this paper only discusses the dimension of public funds per student, but fails to discuss the relationship between investment in education infrastructure, personnel funds and other financial funds and adolescent health; On the other hand, as for the specific mechanism that the financial investment in compulsory education affects adolescents' health, this paper only pays attention to the intermediary effects such as family health investment, school sports facilities and adolescents' social capital, but fails to make extensive and in-depth analysis on other dimensions such as diet and health behavior. In the future, it is necessary to supplement and improve the above problems with richer data.

## Conclusion

In the context of declining fertility rate and deepening aging, the Chinese government hopes to improve adolescent health by increasing financial investment in compulsory education, thereby achieving the goal of population quality replacing population quantity. The results of this study show that the increase in financial investment in compulsory education can not only improve the health of adolescents, but also have a greater impact on vulnerable groups, which is conducive to promoting health equity.

## Supplementary Information

**Additional file 1.**

**Additional file 2.**

## Data Availability

The study utilizes secondary sources of data that are freely available in the public domain through http://cnsda.ruc.edu.cn/index.php?r=projects/view&id=72810330. Those who wish to access the data may register at the above link and thereafter can download the required data free of cost.

## Footnotes

1 http://www.gov.cn/gongbao/content/2018/content_5266234.htm

2 The marital status of parents refers to the current marital status of parents, and whether parents quarrel or not refers to whether they quarrel now or in the past. Although parents may have divorced now, they may have quarreled before, so there is no contradiction between them.

3 Data on age, gender, hukou, whether they are in love or not, whether the only child, parents' marital status, whether parents quarrel are provided by adolescents; Cognitive ability was obtained by investigators' test; data on mother education, family economic status and community characteristics are provided by parents.

4 Considering that the data includes both school and individual levels, according to the reviewers' opinions, it is more appropriate to use the HLM model. However, the null models test results show that the intra-group correlation coefficient (ICC) is less than the critical value of 0.059, so this model is not used in this paper. However, the HLM model results are still robust. The detailed results are shown in the Additional file 1 appendix.
