# Supplementary material for: Evaluating the influence of financial investment in compulsory education on the health of Chinese adolescents: a novel approach
Source: BMC Public Health. 2022 Sep 12;22:1725. doi: 10.1186/s12889-022-14125-5 (PMC9465893; doi:10.1186/s12889-022-14125-5)
Supplement: Supplementary file 1 — Additional file 1. [file 12889_2022_14125_MOESM1_ESM.rtf]

Table Appendix. HLM model
Dep. variables	£¨1£©	£¨2£©	£¨3£©	£¨4£©	
Explanatory variables	Self-rated health	Illness frequency	Sick leave days	Depression	
Main explanatory variable					
Financial investment	0.015**	-0.027**	-0.193***	-0.164***	
	(0.008)	(0.012)	(0.063)	(0.062)	
Individual characteristics					
Age	-0.005	-0.028	0.175*	0.218	
	(0.022)	(0.025)	(0.103)	(0.167)	
Boy	0.105***	-0.270***	0.132	-0.300*	
	(0.032)	(0.034)	(0.126)	(0.169)	
Agriculture	0.016	-0.090**	0.283*	-0.176	
	(0.033)	(0.040)	(0.147)	(0.245)	
Cognitive ability	-0.067***	0.071***	-0.218***	-0.240*	
	(0.016)	(0.019)	(0.082)	(0.133)	
Early health	0.502***	-0.316***	-0.623***	-1.286***	
	(0.021)	(0.024)	(0.090)	(0.111)	
Love	-0.085*	0.036	0.882***	2.857***	
	(0.046)	(0.057)	(0.238)	(0.344)	
Family characteristics					
Parents married	0.091*	0.051	-0.631**	-0.669*	
	(0.050)	(0.078)	(0.296)	(0.403)	
Only child	0.032	0.015	0.099	-0.687***	
	(0.033)	(0.043)	(0.153)	(0.228)	
Parents quarrel	-0.258***	0.314***	0.005	3.949***	
	(0.053)	(0.056)	(0.192)	(0.349)	
Mother's education	-0.001	0.003	0.215**	-0.021	
	(0.016)	(0.020)	(0.105)	(0.125)	
Family economic status	0.154***	-0.055*	0.063	-0.884***	
	(0.025)	(0.030)	(0.203)	(0.193)	
Community characteristics					
Health environment	0.098***	-0.054*	-0.235*	-0.799***	
	(0.025)	(0.028)	(0.123)	(0.173)	
School characteristics					
School level	-0.013	0.023	-0.022	0.552***	
	(0.026)	(0.027)	(0.095)	(0.198)	
County (District) FE	Y	Y	Y	Y	
Wald statistic	3340.72	761.56	2125.88	3790.74	
Note: Values in parentheses are the cluster robust standard error. *p<0.1, **p<0.05, ***p<0.01. Due to space limitations, the cut point is omitted.
