# Supplementary material for: Evaluating the influence of financial investment in compulsory education on the health of Chinese adolescents: a novel approach
Source: BMC Public Health. 2022 Sep 12;22:1725. doi: 10.1186/s12889-022-14125-5 (PMC9465893; doi:10.1186/s12889-022-14125-5)
Supplement: Supplementary file 2 — Additional file 2. [file 12889_2022_14125_MOESM2_ESM.doc]

**Table Appendix. MI** model

| **Dep. variables** | **（1）** | **（2）** | **（3）** | **（4）** |
| --- | --- | --- | --- | --- |
| **Explanatory variables** | **Self-rated health** | **Illness frequency** | **Sick leave days** | **Depression** |
| **Main explanatory variable** |  |  |  |  |
| Financial investment | 0.016^*^ | -0.024^**^ | -0.261^***^ | -0.151^*^ |
|  | (0.009) | (0.012) | (0.073) | (0.081) |
| **Individual characteristics** |  |  |  |  |
| Age | -0.011 | -0.027 | 0.254^**^ | 0.236^*^ |
|  | (0.019) | (0.021) | (0.124) | (0.140) |
| Boy | 0.123^***^ | -0.267^***^ | 0.148 | -0.558^***^ |
|  | (0.024) | (0.029) | (0.134) | (0.174) |
| Agriculture | -0.016 | -0.007^**^ | 0.107 | -0.068 |
|  | (0.028) | (0.035) | (0.181) | (0.231) |
| Cognitive ability | -0.049^***^ | 0.051^***^ | -0.273^***^ | -0.373^***^ |
|  | (0.015) | (0.017) | (0.086) | (0.117) |
| Love | -0.051 | 0.056 | 1.085^***^ | 3.021^***^ |
|  | (0.0436) | (0.046) | (0.313) | (0.308) |
| **Family characteristics** |  |  |  |  |
| Parents married | 0.141^***^ | 0.010 | -0.901^***^ | -0.853^**^ |
|  | (0.042) | (0.055) | (0.316) | (0.331) |
| Only child | 0.008 | 0.065^*^ | 0.066 | -0.423^**^ |
|  | (0.028) | (0.034) | (0.155) | (0.201) |
| Parents quarrel | -0.299^***^ | 0.329^***^ | 0.299 | 4.005^***^ |
|  | (0.043) | (0.052) | (0.265) | (0.325) |
| Mother's education | 0.008 | -0.003 | 0.151 | -0.069 |
|  | (0.014) | (0.016) | (0.095) | (0.096) |
| Family economic status | 0.217^***^ | -0.090^***^ | 0.009 | -0.989^***^ |
|  | (0.023) | (0.024) | (0.174) | (0.166) |
| **Community characteristics** |  |  |  |  |
| Health environment | 0.121^***^ | -0.059^**^ | -0.316^**^ | -0.850^***^ |
|  | (0.020) | (0.025) | (0.152) | (0.150) |
| **School characteristics** |  |  |  |  |
| School level | -0.015 | 0.011 | -0.029 | 0.601^***^ |
|  | (0.019) | (0.019) | (0.089) | (0.162) |
| Constant | - | - | 2.983 | 24.494^***^ |
|  | - | - | (2.195) | (2.454) |
| County (District) FE | Y | Y | Y | Y |
| Observations | 9229 | 9229 | 9229 | 9229 |
| F statistic | 16.75 | 6.77 | 4.82 | 16.39 |

Note: Values in parentheses are the cluster robust standard error. *p<0.1, **p<0.05, ***p<0.01. Due to space limitations, the cut point is omitted.
